# Methyl 3′-benzyl-4′-(2-chloro­phen­yl)-1′-methyl-2-oxo­spiro­[indoline-3,2′-pyrrolidine]-3′-carboxyl­ate

**DOI:** 10.1107/S1600536813018424

**Published:** 2013-07-06

**Authors:** T. Anuradha, A. Devaraj, P. R. Seshadri, M. Bakthadoss

**Affiliations:** a Post Graduate & Research Department of Physics, Agurchand Manmull Jain College, Chennai 600 114, India; b Department of Organic Chemistry, University of Madras, Guindy Campus, Chennai 600 025, India

## Abstract

In the title compound, C_27_H_25_ClN_2_O_3_, the methyl­pyrrolidine ring adopts an envelope conformation with the N atom at the flap. The mean plane of the pyrrolidine ring makes dihedral angles of 82.1 (1), 84.4 (1) and 79.8 (1)°, respectively, with the adjacent benzene ring, the mean plane of the indoline ring system and the phenyl ring. The mol­ecular structure is stabilized by intra­molecular C—H⋯O hydrogen bonds. In the crystal, mol­ecules are linked into chains along [101] by N—H⋯O hydrogen bonds. C—H⋯π inter­actions are observed between the chains.

## Related literature

For the biological activity of pyrrolidine-containing compounds and their use in catalysis, see: Witherup *et al.* (1995 ▶). For the biological activity of oxindole derivatives, see: Glover *et al.* (1998 ▶). For puckering and asymmetry parameters, see: Cremer & Pople (1975 ▶); Nardelli (1983 ▶).
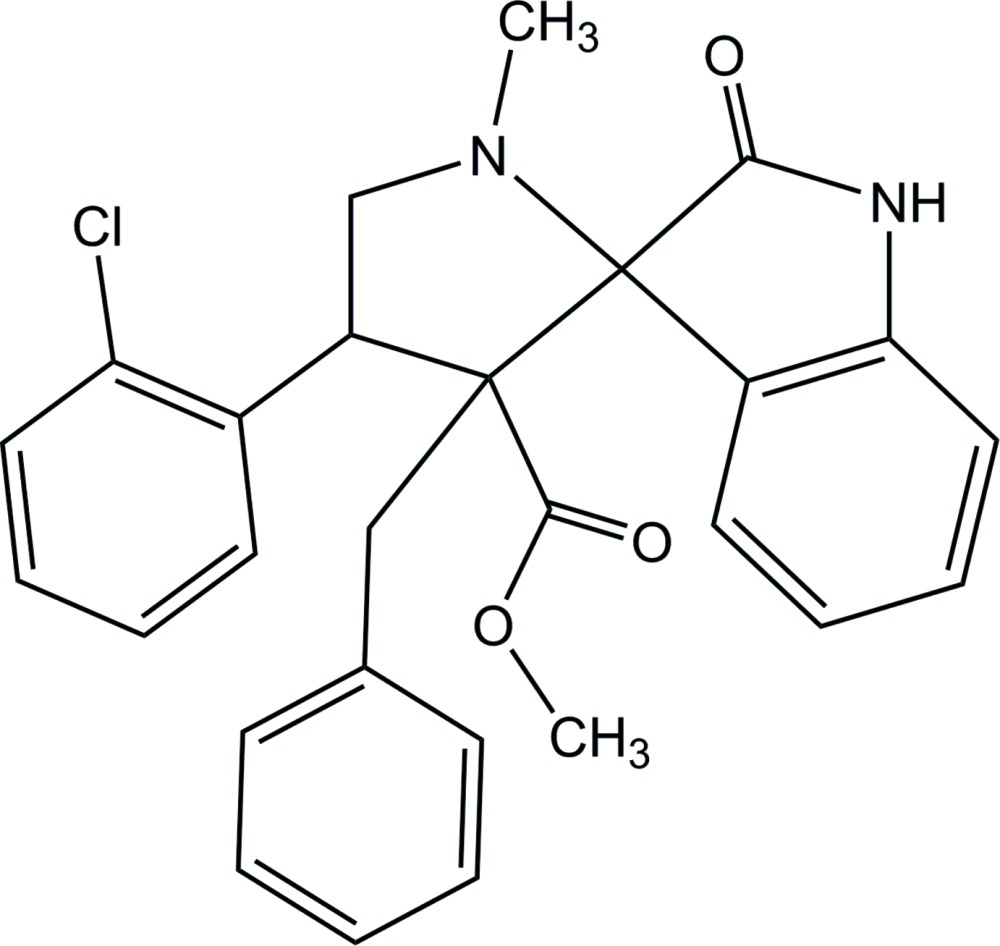


## Experimental

### 

#### Crystal data

C_27_H_25_ClN_2_O_3_
*M* *_r_* = 460.94
Monoclinic, 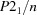

*a* = 13.0887 (6) Å
*b* = 14.0869 (7) Å
*c* = 13.3521 (7) Å
β = 113.524 (2)°
*V* = 2257.25 (19) Å^3^
*Z* = 4
Mo *K*α radiation
μ = 0.20 mm^−1^
*T* = 293 K
0.30 × 0.20 × 0.20 mm

#### Data collection

Bruker Kappa APEXII CCD diffractometer
Absorption correction: multi-scan (*SADABS*; Bruker, 2004 ▶) *T*_min_ = 0.953, *T*_max_ = 0.960
29103 measured reflections
6735 independent reflections
4875 reflections with *I* > 2σ(*I*)
*R*_int_ = 0.028

#### Refinement

*R*[*F* ^2^ > 2σ(*F* ^2^)] = 0.044
*wR*(*F* ^2^) = 0.122
*S* = 0.98
6735 reflections
298 parameters
H-atom parameters constrained
Δρ_max_ = 0.33 e Å^−3^
Δρ_min_ = −0.28 e Å^−3^

### 

Data collection: *APEX2* (Bruker, 2008 ▶); cell refinement: *SAINT* (Bruker, 2008 ▶); data reduction: *SAINT*; program(s) used to solve structure: *SHELXS97* (Sheldrick, 2008 ▶); program(s) used to refine structure: *SHELXL97* (Sheldrick, 2008 ▶); molecular graphics: *ORTEP-3 for Windows* (Farrugia, 2012 ▶); software used to prepare material for publication: *SHELXL97*, *PLATON* (Spek, 2009 ▶) and *publCIF* (Westrip, 2010 ▶).

## Supplementary Material

Crystal structure: contains datablock(s) I, global. DOI: 10.1107/S1600536813018424/is5288sup1.cif

Structure factors: contains datablock(s) I. DOI: 10.1107/S1600536813018424/is5288Isup2.hkl

Supplementary material file. DOI: 10.1107/S1600536813018424/is5288Isup3.cml

Additional supplementary materials:  crystallographic information; 3D view; checkCIF report

## Figures and Tables

**Table 1:** Hydrogen-bond geometry (Å, °) *Cg*3 is the centroid of the C11–C16 ring.

*D*—H⋯*A*	*D*—H	H⋯*A*	*D*⋯*A*	*D*—H⋯*A*
N2—H2*A*⋯O2^i^	0.86	2.06	2.9004 (15)	164
C5—H5⋯O1	0.93	2.31	3.168 (2)	153
C18—H18*A*⋯O1	0.97	2.54	3.2469 (18)	130
C24—H24⋯O3	0.93	2.47	3.1650 (19)	132
C23—H23⋯*Cg*3^ii^	0.93	2.77	3.600 (2)	149

Symmetry codes: (i) 
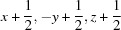
; (ii) 
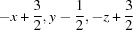
.

**Fig. 1.**
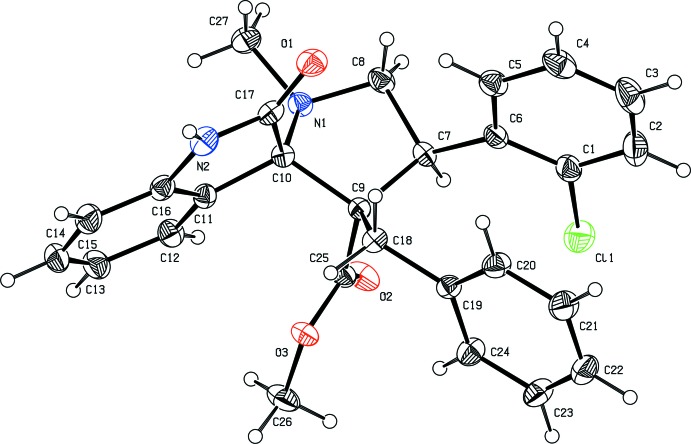
Molecular structure of the title compound, showing the atom-numbering scheme with 30% probability displacement ellipsoids. H atoms are shown as spheres of arbitrary radius.

**Fig. 2.**
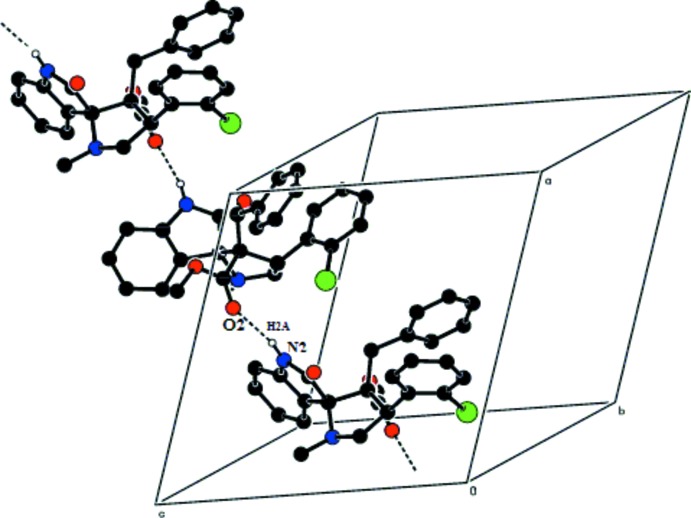
A view of packing of the molecules with hydrogen bonds (dashed lines).

## supplementary crystallographic information

### Comment

Pyrrolidine-containing compounds are of significant importance because of their biological activities and widespread employment in catalysis (Witherup *et al.*, 1995). Oxindole derivatives are of importance in the total synthesis of indole and oxindole alkaloids such as potent inhibitors of monoamine oxidase (MAO) in human urine and rat tissues (Glover *et al.*, 1998). We report herein the crystal structure of the title compound.

In the molecule the pyrrolidine ring (N1/C8–C10) adopts an envelope conformation with N1 atom located at the flap position having asymmetry parameter (Nardelli, 1983) ΔC_S_ (N1) = 5.21 (2) Å and with the puckering parameters (Cremer and Pople, 1975) q2 = 0.396 (2) Å and Φ2 = 172.6 (3)°. The sum of bond angles around N1 of the pyrrolidine ring [336.8 (1)°] is in accordance with *sp*^3^ hybridization. The bond length C17—O1 = 1.210 (1) Å indicates a keto group in the indoline. The pyrrolidine ring (N1/C8–C10) is almost equatorial with indoline (C10–C17/N2), chlorophenyl (C1–C6/Cl1) and phenyl (C19–C24) rings by making dihedral angles of 84.4 (1), 82.1 (1) and 79.8 (1)°, respectively. The indoline ring (C10–C17/N2) makes dihedral angles of 39.5 (2) and 61.2 (1)° with chlorophenyl(C1–C6/Cl1) and the phenyl (C19–C24) rings, respectively.

The structure is stabilized by an intermolecular N—H···O hydrogen bond and C—H···O intramolecular hydrogen bonds. The crystal structure is further consolidated by C—H ···*Cg*3 interactions, where *Cg*3 is the centroid of C11–C16 ring.

### Experimental

A mixture of (*E*)-methyl 2-benzyl-3-(2-chlorophenyl)acrylate (2 mmol, 0.58 g), isatin (2 mmol, 0.29 g) and sarcosine (2 mmol, 0.18 g) in acetonitrile (8 ml) was refluxed for about 10 h. After the completion of the reaction as indicated by TLC, the reaction mixture was concentrated. Then the resulting crude mass was diluted with water (10 ml) and extracted with ethyl acetate (3×10 ml). The combined organic layers were washed with brine (2×10 ml) and dried overanhydrous Na_2_SO_4_. The organic layer was concentrated and the crude sample was purified by column chromatography on silica gel (Acme 100–200 mesh), using ethyl acetate:hexane (1:4) to afford the title compound as a colourless solid in 72% yield.

### Refinement

Hydrogen atoms were positioned geometrically and allowed to ride on their parent atoms, with C—H = 0.93–0.97 Å and N—H = 0.86 Å, and with *U*_iso_(H) = 1.5*U*_eq_(C) for methyl H atoms and 1.2*U*_eq_(C or N) for other H atoms.

### Crystal data

**Table:**

C_27_H_25_ClN_2_O_3_	*F*(000) = 968
*M**_r_* = 460.94	*D*_x_ = 1.356 Mg m^−^^3^
Monoclinic, *P*2_1_/*n*	Mo *K*α radiation, λ = 0.71073 Å
Hall symbol: -P 2yn	Cell parameters from 6735 reflections
*a* = 13.0887 (6) Å	θ = 2.2–30.2°
*b* = 14.0869 (7) Å	µ = 0.20 mm^−^^1^
*c* = 13.3521 (7) Å	*T* = 293 K
β = 113.524 (2)°	Block, colourless
*V* = 2257.25 (19) Å^3^	0.30 × 0.20 × 0.20 mm
*Z* = 4	

### Data collection

**Table:**

Bruker Kappa APEXII CCD diffractometer	6735 independent reflections
Radiation source: fine-focus sealed tube	4875 reflections with *I* > 2σ(*I*)
Graphite monochromator	*R*_int_ = 0.028
ω and φ scan	θ_max_ = 30.3°, θ_min_ = 2.2°
Absorption correction: multi-scan (*SADABS*; Bruker, 2004)	*h* = −18→16
*T*_min_ = 0.953, *T*_max_ = 0.960	*k* = −19→19
29103 measured reflections	*l* = −18→16

### Refinement

**Table:**

Refinement on *F*^2^	Primary atom site location: structure-invariant direct methods
Least-squares matrix: full	Secondary atom site location: difference Fourier map
*R*[*F*^2^ > 2σ(*F*^2^)] = 0.044	Hydrogen site location: inferred from neighbouring sites
*wR*(*F*^2^) = 0.122	H-atom parameters constrained
*S* = 0.98	*w* = 1/[σ^2^(*F*_o_^2^) + (0.0529*P*)^2^ + 0.8661*P*] where *P* = (*F*_o_^2^ + 2*F*_c_^2^)/3
6735 reflections	(Δ/σ)_max_ = 0.001
298 parameters	Δρ_max_ = 0.33 e Å^−^^3^
0 restraints	Δρ_min_ = −0.28 e Å^−^^3^

### Special details

**Table:**

Geometry. All e.s.d.'s (except the e.s.d. in the dihedral angle between two l.s. planes) are estimated using the full covariance matrix. The cell e.s.d.'s are taken into account individually in the estimation of e.s.d.'s in distances, angles and torsion angles; correlations between e.s.d.'s in cell parameters are only used when they are defined by crystal symmetry. An approximate (isotropic) treatment of cell e.s.d.'s is used for estimating e.s.d.'s involving l.s. planes.
Refinement. Refinement of *F*^2^ against ALL reflections. The weighted *R*-factor *wR* and goodness of fit *S* are based on *F*^2^, conventional *R*-factors *R* are based on *F*, with *F* set to zero for negative *F*^2^. The threshold expression of *F*^2^ > σ(*F*^2^) is used only for calculating *R*-factors(gt) *etc*. and is not relevant to the choice of reflections for refinement. *R*-factors based on *F*^2^ are statistically about twice as large as those based on *F*, and *R*- factors based on ALL data will be even larger.

### Fractional atomic coordinates and isotropic or equivalent isotropic displacement parameters (Å^2^)

**Table:**

	*x*	*y*	*z*	*U*_iso_*/*U*_eq_	
Cl1	0.65520 (4)	0.17226 (3)	0.69233 (3)	0.05286 (13)	
O1	0.87657 (9)	0.41364 (8)	1.12278 (9)	0.0420 (3)	
O2	0.59975 (8)	0.11329 (8)	0.94843 (9)	0.0434 (3)	
O3	0.75502 (8)	0.06272 (7)	1.08175 (8)	0.0335 (2)	
N1	0.64083 (9)	0.32985 (8)	1.04601 (9)	0.0297 (2)	
N2	0.90123 (10)	0.31908 (9)	1.26947 (10)	0.0366 (3)	
H2A	0.9655	0.3395	1.3133	0.044*	
C1	0.74514 (13)	0.26845 (11)	0.74148 (12)	0.0377 (3)	
C2	0.79808 (15)	0.30118 (15)	0.67659 (14)	0.0517 (4)	
H2	0.7841	0.2729	0.6094	0.062*	
C3	0.87159 (16)	0.37590 (16)	0.71259 (16)	0.0566 (5)	
H3	0.9073	0.3983	0.6694	0.068*	
C4	0.89246 (15)	0.41751 (13)	0.81149 (16)	0.0489 (4)	
H4	0.9430	0.4674	0.8360	0.059*	
C5	0.83802 (13)	0.38500 (11)	0.87492 (13)	0.0387 (3)	
H5	0.8519	0.4144	0.9415	0.046*	
C6	0.76294 (11)	0.30942 (10)	0.84199 (11)	0.0313 (3)	
C7	0.69843 (11)	0.27784 (10)	0.90838 (10)	0.0286 (3)	
H7	0.6372	0.2379	0.8602	0.034*	
C8	0.64501 (12)	0.36094 (11)	0.94391 (12)	0.0348 (3)	
H8A	0.6898	0.4179	0.9548	0.042*	
H8B	0.5707	0.3737	0.8896	0.042*	
C9	0.76153 (10)	0.21839 (9)	1.01578 (10)	0.0248 (2)	
C10	0.74498 (10)	0.28097 (9)	1.10802 (10)	0.0262 (3)	
C11	0.74272 (11)	0.22971 (10)	1.20696 (10)	0.0289 (3)	
C12	0.66310 (12)	0.17276 (11)	1.21911 (12)	0.0364 (3)	
H12	0.5973	0.1591	1.1595	0.044*	
C13	0.68229 (15)	0.13580 (12)	1.32176 (14)	0.0441 (4)	
H13	0.6293	0.0967	1.3307	0.053*	
C14	0.77965 (16)	0.15694 (13)	1.41035 (13)	0.0468 (4)	
H14	0.7919	0.1306	1.4780	0.056*	
C15	0.85906 (14)	0.21627 (12)	1.40071 (12)	0.0444 (4)	
H15	0.9239	0.2313	1.4607	0.053*	
C16	0.83831 (11)	0.25255 (10)	1.29825 (11)	0.0325 (3)	
C17	0.84848 (11)	0.34756 (10)	1.16400 (12)	0.0306 (3)	
C18	0.88559 (10)	0.19699 (10)	1.04371 (11)	0.0290 (3)	
H18A	0.9254	0.2569	1.0590	0.035*	
H18B	0.9140	0.1602	1.1107	0.035*	
C19	0.91513 (11)	0.14448 (10)	0.95948 (11)	0.0287 (3)	
C20	0.99679 (13)	0.18304 (11)	0.93025 (13)	0.0377 (3)	
H20	1.0299	0.2402	0.9612	0.045*	
C21	1.03006 (16)	0.13863 (12)	0.85642 (15)	0.0476 (4)	
H21	1.0844	0.1662	0.8375	0.057*	
C22	0.98273 (15)	0.05323 (13)	0.81053 (14)	0.0474 (4)	
H22	1.0050	0.0229	0.7607	0.057*	
C23	0.90285 (14)	0.01356 (12)	0.83900 (14)	0.0443 (4)	
H23	0.8708	−0.0441	0.8085	0.053*	
C24	0.86929 (12)	0.05847 (11)	0.91287 (13)	0.0380 (3)	
H24	0.8150	0.0304	0.9316	0.046*	
C25	0.69557 (11)	0.12675 (9)	1.00893 (10)	0.0277 (3)	
C26	0.69898 (15)	−0.02398 (12)	1.08696 (15)	0.0484 (4)	
H26A	0.7497	−0.0646	1.1422	0.073*	
H26B	0.6735	−0.0556	1.0175	0.073*	
H26C	0.6364	−0.0096	1.1046	0.073*	
C27	0.60968 (14)	0.40408 (12)	1.10432 (14)	0.0427 (4)	
H27A	0.5402	0.4319	1.0568	0.064*	
H27B	0.6664	0.4521	1.1276	0.064*	
H27C	0.6018	0.3772	1.1670	0.064*	

### Atomic displacement parameters (Å^2^)

**Table:**

	*U*^11^	*U*^22^	*U*^33^	*U*^12^	*U*^13^	*U*^23^
Cl1	0.0585 (3)	0.0513 (3)	0.0387 (2)	−0.0011 (2)	0.00890 (18)	−0.00687 (17)
O1	0.0440 (6)	0.0340 (6)	0.0505 (6)	−0.0111 (5)	0.0216 (5)	−0.0061 (5)
O2	0.0317 (5)	0.0397 (6)	0.0438 (6)	−0.0094 (4)	−0.0005 (4)	0.0098 (5)
O3	0.0341 (5)	0.0279 (5)	0.0342 (5)	0.0001 (4)	0.0093 (4)	0.0072 (4)
N1	0.0268 (5)	0.0312 (6)	0.0313 (5)	0.0051 (4)	0.0118 (4)	0.0042 (4)
N2	0.0278 (6)	0.0421 (7)	0.0344 (6)	−0.0044 (5)	0.0066 (5)	−0.0083 (5)
C1	0.0384 (7)	0.0406 (8)	0.0326 (7)	0.0098 (6)	0.0128 (6)	0.0075 (6)
C2	0.0536 (10)	0.0701 (12)	0.0376 (8)	0.0157 (9)	0.0248 (7)	0.0097 (8)
C3	0.0517 (10)	0.0732 (13)	0.0583 (11)	0.0097 (10)	0.0361 (9)	0.0239 (10)
C4	0.0425 (9)	0.0457 (9)	0.0666 (11)	0.0013 (7)	0.0302 (8)	0.0166 (8)
C5	0.0391 (8)	0.0352 (8)	0.0454 (8)	0.0001 (6)	0.0206 (6)	0.0054 (6)
C6	0.0327 (7)	0.0311 (7)	0.0321 (6)	0.0052 (5)	0.0150 (5)	0.0079 (5)
C7	0.0278 (6)	0.0301 (7)	0.0263 (6)	0.0000 (5)	0.0092 (5)	0.0051 (5)
C8	0.0325 (7)	0.0354 (7)	0.0378 (7)	0.0077 (6)	0.0154 (6)	0.0103 (6)
C9	0.0240 (6)	0.0249 (6)	0.0244 (5)	−0.0017 (5)	0.0083 (4)	0.0003 (5)
C10	0.0235 (6)	0.0277 (6)	0.0268 (6)	−0.0016 (5)	0.0095 (5)	−0.0004 (5)
C11	0.0276 (6)	0.0321 (7)	0.0272 (6)	0.0031 (5)	0.0110 (5)	0.0002 (5)
C12	0.0335 (7)	0.0399 (8)	0.0375 (7)	−0.0009 (6)	0.0160 (6)	0.0022 (6)
C13	0.0510 (9)	0.0425 (9)	0.0477 (9)	0.0033 (7)	0.0292 (7)	0.0090 (7)
C14	0.0625 (11)	0.0471 (9)	0.0342 (7)	0.0154 (8)	0.0226 (7)	0.0112 (7)
C15	0.0476 (9)	0.0493 (9)	0.0290 (7)	0.0111 (7)	0.0075 (6)	0.0009 (6)
C16	0.0305 (7)	0.0349 (7)	0.0297 (6)	0.0054 (5)	0.0097 (5)	−0.0029 (5)
C17	0.0272 (6)	0.0297 (7)	0.0363 (7)	−0.0017 (5)	0.0142 (5)	−0.0081 (5)
C18	0.0238 (6)	0.0312 (7)	0.0305 (6)	−0.0006 (5)	0.0094 (5)	−0.0034 (5)
C19	0.0251 (6)	0.0291 (6)	0.0303 (6)	0.0036 (5)	0.0094 (5)	0.0001 (5)
C20	0.0384 (8)	0.0321 (7)	0.0474 (8)	−0.0022 (6)	0.0223 (6)	−0.0032 (6)
C21	0.0541 (10)	0.0431 (9)	0.0612 (10)	−0.0035 (8)	0.0395 (9)	−0.0016 (8)
C22	0.0583 (10)	0.0454 (9)	0.0493 (9)	0.0028 (8)	0.0327 (8)	−0.0072 (7)
C23	0.0485 (9)	0.0389 (8)	0.0475 (9)	−0.0044 (7)	0.0213 (7)	−0.0140 (7)
C24	0.0361 (7)	0.0379 (8)	0.0431 (8)	−0.0070 (6)	0.0190 (6)	−0.0084 (6)
C25	0.0280 (6)	0.0271 (6)	0.0264 (6)	−0.0009 (5)	0.0091 (5)	0.0012 (5)
C26	0.0569 (10)	0.0329 (8)	0.0510 (9)	−0.0063 (7)	0.0168 (8)	0.0121 (7)
C27	0.0445 (9)	0.0393 (8)	0.0491 (9)	0.0113 (7)	0.0237 (7)	0.0012 (7)

### Geometric parameters (Å, º)

**Table:**

Cl1—C1	1.7424 (18)	C10—C17	1.5702 (18)
O1—C17	1.2108 (17)	C11—C12	1.375 (2)
O2—C25	1.2053 (16)	C11—C16	1.3918 (18)
O3—C25	1.3269 (15)	C12—C13	1.392 (2)
O3—C26	1.4406 (18)	C12—H12	0.9300
N1—C8	1.4536 (18)	C13—C14	1.381 (3)
N1—C10	1.4550 (16)	C13—H13	0.9300
N1—C27	1.4556 (19)	C14—C15	1.379 (3)
N2—C17	1.3582 (19)	C14—H14	0.9300
N2—C16	1.399 (2)	C15—C16	1.382 (2)
N2—H2A	0.8600	C15—H15	0.9300
C1—C2	1.387 (2)	C18—C19	1.5194 (19)
C1—C6	1.392 (2)	C18—H18A	0.9700
C2—C3	1.377 (3)	C18—H18B	0.9700
C2—H2	0.9300	C19—C24	1.385 (2)
C3—C4	1.369 (3)	C19—C20	1.387 (2)
C3—H3	0.9300	C20—C21	1.377 (2)
C4—C5	1.384 (2)	C20—H20	0.9300
C4—H4	0.9300	C21—C22	1.380 (3)
C5—C6	1.396 (2)	C21—H21	0.9300
C5—H5	0.9300	C22—C23	1.367 (2)
C6—C7	1.5146 (19)	C22—H22	0.9300
C7—C8	1.533 (2)	C23—C24	1.383 (2)
C7—C9	1.5794 (17)	C23—H23	0.9300
C7—H7	0.9800	C24—H24	0.9300
C8—H8A	0.9700	C26—H26A	0.9600
C8—H8B	0.9700	C26—H26B	0.9600
C9—C25	1.5353 (18)	C26—H26C	0.9600
C9—C18	1.5438 (18)	C27—H27A	0.9600
C9—C10	1.5980 (18)	C27—H27B	0.9600
C10—C11	1.5162 (18)	C27—H27C	0.9600
			
C25—O3—C26	116.79 (11)	C14—C13—C12	120.31 (16)
C8—N1—C10	107.39 (10)	C14—C13—H13	119.8
C8—N1—C27	114.12 (12)	C12—C13—H13	119.8
C10—N1—C27	115.75 (11)	C15—C14—C13	121.51 (15)
C17—N2—C16	111.80 (11)	C15—C14—H14	119.2
C17—N2—H2A	124.1	C13—C14—H14	119.2
C16—N2—H2A	124.1	C14—C15—C16	117.31 (15)
C2—C1—C6	122.10 (16)	C14—C15—H15	121.3
C2—C1—Cl1	117.02 (14)	C16—C15—H15	121.3
C6—C1—Cl1	120.89 (12)	C15—C16—C11	122.29 (15)
C3—C2—C1	119.34 (17)	C15—C16—N2	127.96 (14)
C3—C2—H2	120.3	C11—C16—N2	109.65 (12)
C1—C2—H2	120.3	O1—C17—N2	125.27 (13)
C4—C3—C2	120.44 (16)	O1—C17—C10	127.14 (13)
C4—C3—H3	119.8	N2—C17—C10	107.58 (12)
C2—C3—H3	119.8	C19—C18—C9	118.04 (10)
C3—C4—C5	119.68 (17)	C19—C18—H18A	107.8
C3—C4—H4	120.2	C9—C18—H18A	107.8
C5—C4—H4	120.2	C19—C18—H18B	107.8
C4—C5—C6	121.96 (16)	C9—C18—H18B	107.8
C4—C5—H5	119.0	H18A—C18—H18B	107.1
C6—C5—H5	119.0	C24—C19—C20	117.43 (13)
C1—C6—C5	116.47 (14)	C24—C19—C18	124.48 (13)
C1—C6—C7	121.63 (13)	C20—C19—C18	118.04 (12)
C5—C6—C7	121.78 (13)	C21—C20—C19	121.54 (15)
C6—C7—C8	112.74 (11)	C21—C20—H20	119.2
C6—C7—C9	118.39 (11)	C19—C20—H20	119.2
C8—C7—C9	105.17 (10)	C20—C21—C22	120.00 (15)
C6—C7—H7	106.6	C20—C21—H21	120.0
C8—C7—H7	106.6	C22—C21—H21	120.0
C9—C7—H7	106.6	C23—C22—C21	119.39 (15)
N1—C8—C7	104.64 (11)	C23—C22—H22	120.3
N1—C8—H8A	110.8	C21—C22—H22	120.3
C7—C8—H8A	110.8	C22—C23—C24	120.50 (15)
N1—C8—H8B	110.8	C22—C23—H23	119.7
C7—C8—H8B	110.8	C24—C23—H23	119.7
H8A—C8—H8B	108.9	C23—C24—C19	121.13 (14)
C25—C9—C18	111.25 (11)	C23—C24—H24	119.4
C25—C9—C7	108.29 (10)	C19—C24—H24	119.4
C18—C9—C7	116.19 (11)	O2—C25—O3	122.21 (12)
C25—C9—C10	105.29 (10)	O2—C25—C9	125.61 (12)
C18—C9—C10	112.00 (10)	O3—C25—C9	112.13 (10)
C7—C9—C10	102.98 (10)	O3—C26—H26A	109.5
N1—C10—C11	112.42 (11)	O3—C26—H26B	109.5
N1—C10—C17	114.99 (11)	H26A—C26—H26B	109.5
C11—C10—C17	100.54 (10)	O3—C26—H26C	109.5
N1—C10—C9	101.95 (10)	H26A—C26—H26C	109.5
C11—C10—C9	117.69 (11)	H26B—C26—H26C	109.5
C17—C10—C9	109.87 (10)	N1—C27—H27A	109.5
C12—C11—C16	119.35 (13)	N1—C27—H27B	109.5
C12—C11—C10	131.30 (12)	H27A—C27—H27B	109.5
C16—C11—C10	109.25 (12)	N1—C27—H27C	109.5
C11—C12—C13	119.14 (14)	H27A—C27—H27C	109.5
C11—C12—H12	120.4	H27B—C27—H27C	109.5
C13—C12—H12	120.4		
			
C6—C1—C2—C3	0.7 (2)	C17—C10—C11—C16	−8.30 (14)
Cl1—C1—C2—C3	−178.80 (14)	C9—C10—C11—C16	110.91 (13)
C1—C2—C3—C4	0.1 (3)	C16—C11—C12—C13	−2.8 (2)
C2—C3—C4—C5	−0.9 (3)	C10—C11—C12—C13	−178.68 (15)
C3—C4—C5—C6	1.0 (3)	C11—C12—C13—C14	0.6 (2)
C2—C1—C6—C5	−0.6 (2)	C12—C13—C14—C15	1.4 (3)
Cl1—C1—C6—C5	178.88 (11)	C13—C14—C15—C16	−1.1 (2)
C2—C1—C6—C7	175.46 (14)	C14—C15—C16—C11	−1.2 (2)
Cl1—C1—C6—C7	−5.07 (19)	C14—C15—C16—N2	174.75 (15)
C4—C5—C6—C1	−0.3 (2)	C12—C11—C16—C15	3.2 (2)
C4—C5—C6—C7	−176.31 (14)	C10—C11—C16—C15	179.89 (13)
C1—C6—C7—C8	−128.67 (14)	C12—C11—C16—N2	−173.40 (13)
C5—C6—C7—C8	47.18 (17)	C10—C11—C16—N2	3.29 (16)
C1—C6—C7—C9	108.02 (15)	C17—N2—C16—C15	−171.99 (15)
C5—C6—C7—C9	−76.13 (17)	C17—N2—C16—C11	4.37 (16)
C10—N1—C8—C7	40.65 (14)	C16—N2—C17—O1	169.08 (14)
C27—N1—C8—C7	170.37 (12)	C16—N2—C17—C10	−9.77 (15)
C6—C7—C8—N1	−150.69 (11)	N1—C10—C17—O1	−47.09 (19)
C9—C7—C8—N1	−20.31 (14)	C11—C10—C17—O1	−168.07 (14)
C6—C7—C9—C25	−126.30 (13)	C9—C10—C17—O1	67.20 (17)
C8—C7—C9—C25	106.70 (12)	N1—C10—C17—N2	131.74 (12)
C6—C7—C9—C18	−0.28 (17)	C11—C10—C17—N2	10.76 (13)
C8—C7—C9—C18	−127.29 (12)	C9—C10—C17—N2	−113.97 (12)
C6—C7—C9—C10	122.52 (12)	C25—C9—C18—C19	67.05 (15)
C8—C7—C9—C10	−4.48 (13)	C7—C9—C18—C19	−57.46 (16)
C8—N1—C10—C11	−169.67 (11)	C10—C9—C18—C19	−175.41 (11)
C27—N1—C10—C11	61.54 (15)	C9—C18—C19—C24	−53.85 (19)
C8—N1—C10—C17	76.09 (14)	C9—C18—C19—C20	128.89 (14)
C27—N1—C10—C17	−52.70 (16)	C24—C19—C20—C21	1.0 (2)
C8—N1—C10—C9	−42.72 (13)	C18—C19—C20—C21	178.49 (15)
C27—N1—C10—C9	−171.51 (11)	C19—C20—C21—C22	−0.7 (3)
C25—C9—C10—N1	−85.91 (11)	C20—C21—C22—C23	0.1 (3)
C18—C9—C10—N1	153.05 (11)	C21—C22—C23—C24	0.2 (3)
C7—C9—C10—N1	27.49 (12)	C22—C23—C24—C19	0.2 (3)
C25—C9—C10—C11	37.55 (14)	C20—C19—C24—C23	−0.8 (2)
C18—C9—C10—C11	−83.49 (14)	C18—C19—C24—C23	−178.06 (14)
C7—C9—C10—C11	150.94 (11)	C26—O3—C25—O2	−1.1 (2)
C25—C9—C10—C17	151.71 (10)	C26—O3—C25—C9	176.38 (12)
C18—C9—C10—C17	30.67 (14)	C18—C9—C25—O2	−146.65 (14)
C7—C9—C10—C17	−94.90 (11)	C7—C9—C25—O2	−17.80 (19)
N1—C10—C11—C12	45.1 (2)	C10—C9—C25—O2	91.82 (16)
C17—C10—C11—C12	167.86 (15)	C18—C9—C25—O3	35.95 (15)
C9—C10—C11—C12	−72.93 (19)	C7—C9—C25—O3	164.80 (11)
N1—C10—C11—C16	−131.09 (12)	C10—C9—C25—O3	−85.58 (13)

### Hydrogen-bond geometry (Å, º)

*Cg*3 is the centroid of the C11–C16 ring.

**Table:**

*D*—H···*A*	*D*—H	H···*A*	*D*···*A*	*D*—H···*A*
N2—H2*A*···O2^i^	0.86	2.06	2.9004 (15)	164
C5—H5···O1	0.93	2.31	3.168 (2)	153
C18—H18*A*···O1	0.97	2.54	3.2469 (18)	130
C24—H24···O3	0.93	2.47	3.1650 (19)	132
C23—H23···*Cg*3^ii^	0.93	2.77	3.600 (2)	149

Symmetry codes: (i) *x*+1/2, −*y*+1/2, *z*+1/2; (ii) −*x*+3/2, *y*−1/2, −*z*+3/2.
